# Quantification of Hepatic Iron Concentration in Chronic Viral Hepatitis: Usefulness of T2-weighted Single-Shot Spin-Echo Echo-Planar MR Imaging

**DOI:** 10.1371/journal.pone.0033868

**Published:** 2012-03-16

**Authors:** Tatsuyuki Tonan, Kiminori Fujimoto, Aliya Qayyum, Takumi Kawaguchi, Atsushi Kawaguchi, Osamu Nakashima, Koji Okuda, Naofumi Hayabuchi, Michio Sata

**Affiliations:** 1 Department of Radiology, Kurume University School of Medicine, Kurume University Hospital, Kurume, Fukuoka, Japan; 2 Center for Diagnostic Imaging, Kurume University Hospital, Kurume, Fukuoka, Japan; 3 Department of Radiology and Biomedical Imaging, University of California San Francisco, San Francisco, California, United States of America; 4 Department of Digestive Disease Information & Research and Department of Internal Medicine, Kurume University School of Medicine, Kurume, Fukuoka, Japan; 5 Biostatics Center, Kurume University School of Medicine, Kurume, Fukuoka, Japan; 6 Department of Clinical Laboratory Medicine, Kurume University Hospital, Kurume, Fukuoka, Japan; 7 Department of Surgery, Department of Medicine, Kurume University School of Medicine, Kurume, Fukuoka, Japan; 8 Division of Gastroenterology, Department of Medicine, Kurume University School of Medicine, Kurume, Fukuoka, Japan; The University of Hong Kong, Hong Kong

## Abstract

**Objective:**

To investigate the usefulness of single-shot spin-echo echo-planar imaging (SSEPI) sequence for quantifying mild degree of hepatic iron stores in patients with viral hepatitis.

**Methods:**

This retrospective study included 34 patients with chronic viral hepatitis/cirrhosis who had undergone histological investigation and magnetic resonance imaging with T2-weighted gradient-recalled echo sequence (T2-GRE) and diffusion-weighted SSEPI sequence with *b*-factors of 0 s/mm^2^ (T2-EPI), 500 s/mm^2^ (DW-EPI-500), and 1000 s/mm^2^ (DW-EPI-1000).

The correlation between the liver-to-muscle signal intensity ratio, which was generated by regions of interest placed in the liver and paraspinous muscles of each sequence image, and the hepatic iron concentration (µmol/g dry liver), which was assessed by spectrophotometry, was analyzed by linear regression using a spline model. Akaike information criterion (AIC) was used to select the optimal model.

**Results:**

Mean ± standard deviation of the hepatic iron concentration quantified by spectrophotometry was 24.6±16.4 (range, 5.5 to 83.2) µmol/g dry liver. DW-EPI correlated more closely with hepatic iron concentration than T2-GRE (R square values: 0.75 for T2-EPI, 0.69 for DW-EPI-500, 0.62 for DW-EPI-1000, and 0.61 for T2-GRE, respectively, all *P<*0.0001). Using the AIC, the regression model for T2-EPI generated by spline model was optimal because of lowest cross validation error.

**Conclusion:**

T2-EPI was sensitive to hepatic iron, and might be a more useful sequence for quantifying mild degree of hepatic iron stores in patients with chronic viral hepatitis.

## Introduction

Abnormalities of iron metabolism are frequently observed in patients with chronic liver diseases such as viral hepatitis, nonalcoholic fatty liver disease, and cirrhosis [1], [2]. Iron excess, which increases oxidative stress via the formation of hydroxyl radicals and other highly reactive oxidizing molecules, leads to hepatotoxicity; it is related to the fibrogenesis and hepatocarcinogenesis associated with chronic viral hepatitis [1], [3].

In recent years, several research groups have reported on the efficacy of iron reduction therapies by phlebotomy [4]–[10]. Yano et al. [6] reported that phlebotomy therapy contributed to improvement of biochemical markers in patients with hepatitis C virus infection. Kato et al. [10] stated that phlebotomy therapy may potentially lower the risk of progression to hepatocellular carcinoma (HCC) in patients with hepatitis C virus infection. Therefore, precise quantification of hepatic iron overload might be beneficial for managing iron reduction therapy in patients with chronic viral hepatitis.

Assessment of body iron stores by measurement of serum ferritin concentration has poor specificity [11]. Liver biopsy, the most reliable method to measure hepatic iron stores, is an invasive procedure. Magnetic resonance imaging (MRI) is sensitive to hepatic iron because iron leads to a decline of MR signal due to T2-shortening effect related to paramagnetic properties. MRI has recently been recognized as a suitable noninvasive technique for quantifying hepatic iron overload [12]. Quantification of hepatic iron overload by MRI is useful in that it obviates the need for invasive liver biopsy and allows for repeat performance.

Generally, it is accepted that gradient-recalled echo (GRE) sequences are the most sensitive sequence to quantify mild degree of hepatic iron overload [13]–[20]. However, many studies evaluating GRE sequence with different echo-time and flip angle report variable results in the quantification of hepatic iron overload. Although the reproducibility of the technique and the quantification algorithm has been validated in various centers, these results are complicated.

Diffusion-weighted (DW) single-shot spin-echo echo-planar imaging (DW-EPI) has become a sequence used routinely in many institutions since the image quality was improved by recent technical progress such as parallel imaging and respiratory triggering [21]–[23]. In previous studies, it was reported that single-shot spin-echo EPI (SSEPI) sequence also had a high susceptibility effect [24], [25].

We postulate that DW-EPI sequence might be superior to GRE sequence for quantifying mild degree of hepatic iron stores. To our knowledge, the investigation of hepatic iron overload by DW-EPI sequence has not been examined. The aim of this study was to investigate the usefulness of SSEPI sequence for quantifying mild degree of hepatic iron stores in patients with viral hepatitis.

## Materials and Methods

### Patients

The institutional review board (the Ethics Committee of Kurume University) approved this retrospective study (Approval No. 09112), which complied with the principles of the Declaration of Helsinki (2008 version). All included patients gave written informed consent to participate.

Our study was targeted at patients with viral chronic hepatitis/cirrhosis and HCC because such patients with chronic liver impairment may have increased liver iron and would have undergone both liver MR imaging and hepatic surgery.

We reviewed the patients who admitted use of both liver specimens and MR images before hepatic surgery at our institution between January 2007 and April 2008 and identified patients who met the following inclusion criteria: (*a*) patients had both chronic viral hepatitis/cirrhosis and HCC; (*b*) patients underwent abdominal MR imaging with T2-weighted GRE sequence and DW-EPI sequence with *b*-factors of 0 s/mm^2^, 500 s/mm^2^, and 1000 s/mm^2^ (these sequences were part of our standard abdominal MR imaging protocol during this period); and (*c*) patients underwent an operation for HCC and received a histopathologic diagnosis of either chronic hepatitis or cirrhosis that was based on findings at surgical resection, performed within a month after MR imaging.

Forty-six patients fulfilled these criteria. Twelve of these 46 patients were excluded on the basis of the following reasons: *(a)* Available imaging data did not correspond to available histopathologic data because of interval surgery (*n* = 5), *(b)* MR studies were incomplete (*n* = 3), *(c)* an artifact was observed on MR images and precluded accurate measurement of signal intensity (*n* = 1), and *(d)* other causes of chronic liver disease such as alcoholic hepatitis (*n* = 2) and non-alcoholic steatohepatitis (*n* = 1). Thirty-four patients formed the final study group (21 men and thirteen women; median age, 65 years; range, 52–83 years). Histopathologic sampling of all patients included in the study was performed after MR imaging (median, 5 days; range, 1–30 days). The cause of chronic liver disease was hepatitis C virus infection (n = 26) or hepatitis B virus infection (n = 8). None of the patients had a clinical diagnosis of hemochromatosis that was based on review of medical records.

### Hepatic iron concentration and histological analysis

A partial hepatic resection was performed in all patients with HCC. For each patient, 50 mg of wet liver tissue was extracted from the surgically removed specimen by a MLS1200 MEGA microwave digestion system (Milestone General Co. Ltd., Kawasaki, Japan) for 1 min at 250 W, 1 min at 0 W, 5 min at 250 W, 5 min 400 W, and 5 min at 500 W. For determination of hepatic iron concentration (µmol/g dry liver), the resulting extracts were analyzed by spectrophotometry with a graphite atomic absorption camera (Polarized Zeeman Atomic Absorption Spectrophotometer, Hitachi, Ltd., Tokyo, Japan) and were converted to the units shown above [26].

For histological analysis, fibrosis stage and necroinflammation grade were evaluated semiquantitatively using the METAVIR scoring system [27]. Fibrosis stage graded on a scale of 0 to 4, as follows: F0 = no fibrosis; F1 = portal fibrosis without septa; F2 = portal fibrosis and few septa; F3 = numerous septa without cirrhosis; and F4 = cirrhosis. The necroinflammatory activity score was graded on a scale of 0 to 3, as follows: A0 = none; A1 = mild; A2 = moderate; A3 = severe. Distribution of steatosis was also retrospectively evaluated as the overall impression of the percentage of fat-containing hepatocytes on hematoxylin and eosin–stained specimens [28], [29]. Steatosis grade was scored on a scale of 0 to 2, as follows: grade 0 = absence of steatosis; grade 1 = steatosis <5%; and grade 2 = steatosis ≥5%.

### MRI technique and analysis

Within one month prior to surgery, MR imaging was performed at field strength of 1.5 T (Magnetom Symphony Advanced; Siemens, Erlangen, Germany) with use of a body phased-array surface coil. A series of DWIs and T2-weighted GRE sequence were obtained using parallel imaging with generalized auto calibrating partially parallel acquisition (GRAPPA) of acceleration factor 2 in all patients. DWI was performed in the transverse plane by respiratory-triggered combining SSEPI sequence with a chemical shift–selective pulse (CHESS). Any antiperistalsis drug was not used.

The imaging parameters for DW-EPI were as follows: repetition time (TR), 2000 msec; echo time (TE), 81 msec; directions of the motion-probing gradient, three orthogonal axes; gradient factor *b* values of 0 sec/mm^2^ (T2-weighted SSEPI, hereafter T2-EPI), 500 sec/mm^2^ (DW-EPI-500), and 1000 sec/mm^2^ (DW-EPI-1000); 2170-Hz per pixel bandwidth; 350-mm field of view; 128×88 rectangular matrixes; 9-mm-thick sections; 1-mm intersection gap; six signals acquired; and acquisition time of approximately 1 minute 30 seconds.

T2-weighted GRE sequence (hereafter, T2-GRE) was performed in the transverse plane by fast low angle shot (FLASH) with one signal acquired during a 22-second breath hold. The imaging parameters for T2-GRE were as follows: TR, 246 msec; TE, 9.5 msec; flip angle (FA), 30°; 350-mm field of view; 9-mm-thick sections; 1-mm intersection gap; 16-number of sections; 256×192 matrix; and 130-Hz per pixel bandwidth.

**Figure 1:**
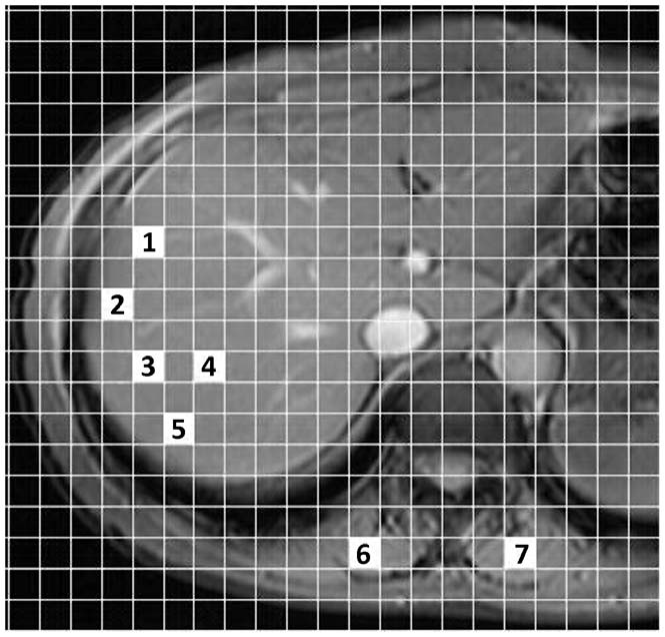
Illustration of the method used to measure regions of interest on an MR image. With use of computer software (developed in-house by the authors), two independent observers freely and easily selected a region of interest by clicking a mesh unit on the right hepatic lobe of an image while avoiding the large vessels, focal hepatic lesions, or artifacts. Seven regions of interest were chosen for liver parenchyma (1–5, total liver ROI area sampled, 500 mm^2^) and paraspinous muscles (6 and 7, total muscle ROI area sampled, 200 mm^2^) in the same slice section of each sequence.

Quantitative image analysis was conducted by measuring the signal intensities of the liver parenchyma and paraspinous muscles. Image analysis was performed by two independent radiologists using plug-in software developed in-house by one of the authors [30], [31] (Figure 1). Five separate regions of interest (ROIs) were carefully placed manually in the anterior and posterior segments of the right hepatic lobe at the level of the porta hepatis (whenever possible) on each sequence; care was taken to avoid focal lesions, major vascular structures, and artifacts such as chemical shifts, magnetic susceptibility, and cardiac motion. Liver signal intensities were recorded as the mean values generated from the five measurements (total liver ROI area sampled, 500 mm^2^). The procedure was repeated to measure muscle signal intensity by placing two separate ROIs on the right and left paraspinous muscles in the same slice section used to measure liver signal intensity; care was taken to avoid artifacts such as chemical shifts, magnetic susceptibility, and motion on each sequence.

Muscle signal intensities were recorded as the mean values generated from the two measurements (total muscle ROI area sampled, 200 mm^2^). We calculated the liver-to-muscle signal intensity ratio (LMR) by dividing mean liver signal intensity by mean muscle signal intensity for each sequence [15].

### Statistical analysis

A Bland-Altman plot was used to analyze the 95% limits of interobserver agreement for the LMR on each sequence [32]. The correlation of the LMR obtained by the two observers on each sequence was determined using the Pearson correlation coefficient (*r*).

The relationship between the LMR on each sequence and hepatic iron concentration was analyzed by means of scatter plots. These results were inspected for linearity and goodness of fit. The relationship between the LMR on each sequence and hepatic iron concentration was modeled by regression techniques using a spline model. Details of spline models are given in the next section.

To investigate effects of each LMR on hepatic iron concentration, we applied the linear models containing not only a main term but also knot terms which play a role as an inflection point. The Akaike information criterion (AIC) was used to evaluate these alternate models [33]. The number and location of knots were determined objectively with the minimum AIC among their prespecified candidates, which were 20, 40, 60, and 80 percentiles of each LMR. To evaluate the predictive accuracy, a leave one out cross validation (CV) error [34] was computed.

The Kruskal–Wallis test was used to determine significant differences in the LMR on each sequence among category classification in each histological finding (i.e. necroinflammation grade, fibrosis stage, and steatosis grade). All analyses were performed using SPSS statistical software (version 12.0 J; SPSS, Inc., Chicago, IL, USA). *P*<0.05 was considered statistically significant.

### Details of the spline models used in statistical analysis

Response and predictor variables are denoted by y and x, respectively. The general form of the univariate (first order) spline model is
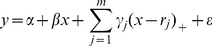
(1)where 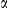, 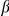, and 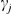 (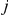 = 1, 2, …, m) are parameters to be estimated, 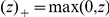, 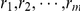 are called knots which play a role as an inflection point, and 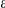 is an error following a normal distribution with mean 0 and a constant variance. Note that in the case of 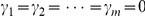 the model can be identified as a simple linear regression model. The parameters in the model (1) are estimated by an ordinary least squares method to minimize squared residuals Q in (2) from samples 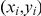 (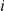 = 1, 2, …, n) from 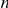 patients.
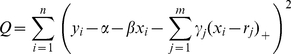
(2)To illustrate the interpretation of parameters in the spline model, we consider the model as with only one knot as in (3). This model contains two lines whose slope and intercept are changed at 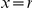.
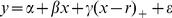
(3)In the range 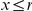, the slope is 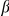 and the intercept is 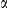. In the other range 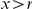, the slope is 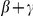 and the intercept is 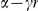.This modeling can be easily implemented by standard software such as SAS, SPSS, and R. Supposing that the data set has two columns corresponding to response (y) and predictor (x) variables, one can add the computed 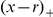 as the third column. Then, the multiple regression model can be applied with the response y and two predictors, x and 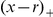. If you want more knots, you can add the corresponding columns and predictors in the regression model.

The essential point in the use of this spline model is to select the number and location of knots. As used in this paper, one choice for candidates for knots is the quantiles for continuous variables taking into account the sample size. Once one specifies the candidates, the problem turns to the variable selection for predictor variables used in the multiple regression model, which can also be implemented by standard software. One effective method is to use information criteria such as AIC. This kind of modeling [35] is useful to investigate the flexible relationship between the response and predictor.

## Results

### Hepatic iron concentration and histological findings

Mean ± SD of the hepatic iron concentration quantified by spectrophotometry was 24.6±16.4 (range, 5.5 to 83.2) µmol/g dry liver. Histological necroinflammation grade was A1 in 21 patients and A2 in 13 patients. Fibrosis stage was F1 in 13 patients, F2 in 4 patients, F3 in 5 patients, and F4 (i.e. cirrhosis) in 12 patients. Steatosis grade was 0 in 14 patients, grade 1 in 11 patients, and grade 2 in 9 patients.

### Interobserver agreement for the LMR on each sequence

**Figure 2:**
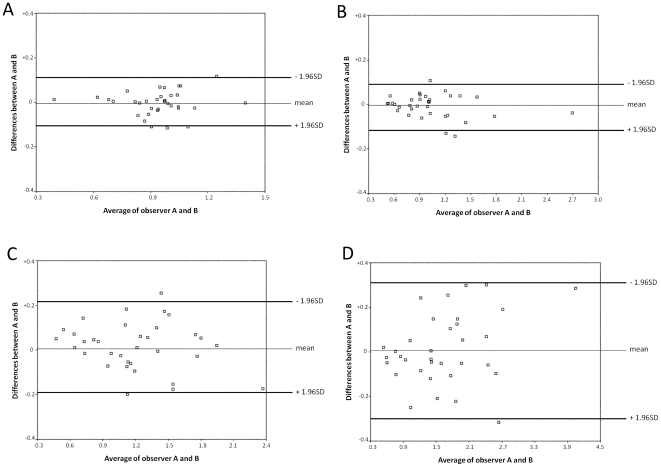
Bland-Altman plots for measurements of T2-GRE (A), T2-EPI (B), DW-EPI-500 (C), and DW-EPI-1000 (D) in liver parenchyma. Each Bland-Altman plots demonstrates good interobserver agreement and lack of proportional bias or fixed bias. The average of the measurements made by the two observers is plotted against the difference between the measurements made by the two observers. The thin lines represent the mean value of all differences between the two observers, and the thick lines represent the 95% limits of agreement. *SD* = standard deviation.

There was no significant difference between measurements made by the two observers for the two parameters; the interclass Pearson correlation coefficients were 0.96 (95% confidence interval [CI]: 0.86, 1.00) for T2-GRE, 0.99 (95% CI: 0.92, 1.00) for T2-EPI, 0.97 (95% CI: 0.85, 1.00) for DW-EPI-500, and 0.98 (95% CI: 0.97, 1.00) for DW-EPI-1000; the mean difference (± standard deviation) was −0.0027±0.054 for T2-GRE, −0.0069±0.052 for T2-EPI, 0.017±0.11 for DW-EPI-500, and 0.013±0.16 for DW-EPI-1000; and the coefficients of repeatability were 0.108 for T2-GRE, 0.105 for T2-EPI, 0.213 for DW-EPI-500, and 0.316 for DW-EPI-1000. Bland-Altman plots with 95% limits of agreement for each sequence are shown in Figure 2. There was no proportional bias or fixed bias in each Bland-Altman plot for the two parameters.

### Correlation between the LMR on each sequence and hepatic iron concentration

**Figure 3:**
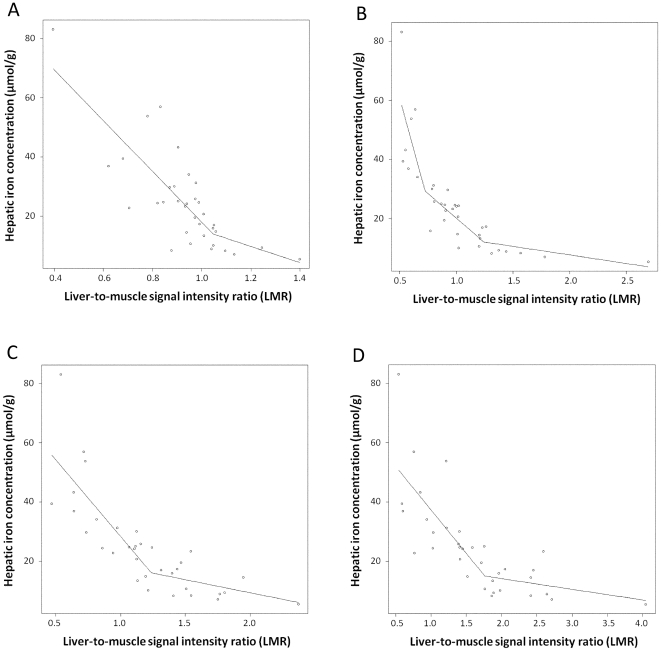
Scatter plots of LMR and hepatic iron concentration (µmol/g dry liver) on T2-GRE (A), T2-EPI (B), DW-EPI-500 (C), and DW-EPI-1000 (D). Correlation between LMR and hepatic iron concentration for linear regression with spline models are shown as solid lines on each sequence. The linear regression model [y = 131.0−139.7×LMR+106.5×(LMR−0.73)_+_+27.4×(LMR−1.24)_+_] on T2-EPI was optimal.

Figure 3 shows results for the line fit by the selected regression model. Created simple regression models to estimate the hepatic iron concentration in each sequence are as follows:
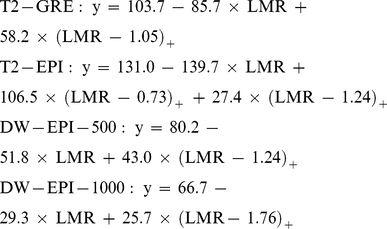
where LMR is the measurement value on each sequence (appendix).

The regression analyses showed an excellent overall negative correlation on each sequence. Particularly, T2-EPI correlated most closely with hepatic iron concentration. R square values on each sequence were as follows: 0.75 for T2-EPI, 0.69 for DW-EPI-500, 0.62 for DW-EPI-1000, and 0.61 for T2-GRE (F-test, *P<*0.0001, respectively).

Using the AIC, the linear regression model on T2-EPI [y = 131.0−139.7×LMR+106.5×(LMR−0.73)_+_+27.4×(LMR−1.24)_+_] was chosen as having the best fit, since it had the lowest CV error. The corresponding CV errors were as follows: 14161.3 for T2-GRE, 11357.4 for T2-EPI, 12220.0 for DW-EPI-500, and 14376.2 for DW-EPI-1000.

### Correlation between the LMR on each sequence and histological findings

No significant differences were found for the LMR on each sequence among category classification of histological findings (i.e. necroinflammation grade, fibrosis stage, and steatosis grade). *P* values (Kruskal-Wallis test) were as follows: (a) necroinflammation grade: *P* = 0.4 for T2-GRE, *P* = 0.89 for T2-EPI, *P* = 0.68 for DW-EPI-500, and *P* = 0.6 for DW-EPI-1000; (b) fibrosis stage: *P* = 0.39 for T2-GRE, *P* = 0.29 for T2-EPI, *P* = 0.19 for DW-EPI-500, and *P* = 0.38 for DW-EPI-1000; (c) steatosis grade: *P* = 0.75 for T2-GRE, *P* = 0.77 for T2-EPI, *P* = 0.69 for DW-EPI-500, and *P* = 0.95 for DW-EPI-1000.

## Discussion

In the present study, we found good correlation between DW-EPI and hepatic iron concentration in patients with chronic viral hepatitis, and also demonstrated that SSEPI sequence was more sensitive than T2-GRE sequence for quantifying small amount of hepatic iron overload; this is in concordance with prior studies reporting a high susceptibility effect with SSEPI sequence [24], [25].

A lot of studies have evaluated the correlation between hepatic iron concentration and MRI measurements [13]–[20]. Particularly, GRE sequences, which are more sensitive to field heterogeneities than spin-echo sequences [15], [16], [18], were used for quantifying mild degree of hepatic iron stores in many studies. It was reported that the best means to evaluate mild degrees of hepatic iron overload was T2-GRE sequences with long TE (i.e. >15 ms) and with low FA (i.e. 20°–30°) [15], [19]. Alternatively, Bonkovsky et al. [18] reported that GRE sequence with shortest TR and TE, which results in a short breath hold time, was useful to minimize motion artifact and other sources of noise. Results from studies of GRE sequence were variable in terms of quantification of hepatic iron overload [13]–[20].

The sensitivity to iron on T2-GRE sequences varies significantly with various different TE and FA [15]. Marked signal loss from proton dephasing will occur at longer TEs, and once signal intensity falls to the level of image noise, inaccuracies in signal intensity measurement can be expected [36]. From these points, in the routine examination, we employed the conventional TE which corresponds to second in-phase on T2-GRE sequence for quantifying mild degree of hepatic iron overload.

SSEPI sequences are very fast and have a high susceptibility effect, but suffer from limited image quality. This is mostly related to limited signal to noise ratio (SNR), especially at higher b-values, and limited spatial resolution, which constitute an obstacle for its widespread use in clinical practice [37]. However, techniques such as parallel imaging and pulse triggering improve image quality of SSEPI sequences by correcting magnetic field heterogeneity [21]–[23]. Recent data showed that respiratory triggering improved the image quality with SNR on SSEPI sequences. This method attempts to avoid motion artifacts prospectively by using respiratory signals to synchronize image acquisition with the patient's breathing cycle and by acquiring the imaging data during the relative quite end expiration phase [38]–[40].

In the present study, we employed SSEPI sequence with techniques such as parallel imaging and respiratory triggering. This sequence, which has the advantage of high susceptibility effects, was useful to assess mild degree of hepatic iron stores in patients with viral hepatitis. Of DW-EPIs, it was suggested that T2-EPI was the most suitable sequence because DW-EPI-500 and DW-EPI-1000 had loss of SNR caused by application of the motion-probing gradients pulse.

In patients with chronic viral hepatitis, steatosis is a common secondary phenomenon. Westphalen et al. [41] reported that iron stores in background liver complicated measurement of steatosis by opposed-phase MR imaging. Alternatively, a recent study reported that concomitant steatosis lowers the diagnostic performance of T2-GRE sequence and chemical shift imaging for quantifying mild degree of hepatic iron stores because intravoxel constructive and destructive interference between fat and water spins due to chemical shift effect of the second kind potentially affect the signal intensity measurements for T2-GRE sequence [36]. Therefore, it might be important to consider the influence of each factor in background liver tissue in the quantification of steatosis and iron stores using MR imaging.

On DW-EPI, we found no significant differences in LMR among histological steatosis grades. Use of fat saturation pulse (i.e., CHESS) on DW-EPIs could eliminate the influence of steatosis, which might support the better utility of this sequence for quantifying mild degree of hepatic iron stores. On the other hand, although previous studies reported that liver fibrosis decreased the diffusion signal [30], [42], [43], no significant differences were found in LMR on DW-EPIs among histological fibrosis stages, which suggest that influence of liver fibrosis to the signal of DW-EPIs was low as a result. The quantification of iron stores by DW-EPIs may have suffered potential influence by fibrosis, which might be one of the reasons that T2-EPI was most accurate sequence for quantifying mild degree of iron stores. Therefore, we recommend the T2-EPI with *b* values of 0 sec/mm^2^, which is not affected to the diffusion signal, for quantifying mild degree of iron stores.

Several limitations of the present study warrant mention. First, the study was conducted retrospectively and sample size was small. Although a major effort was made to exclude sample bias, there was limited sample size for examination of liver iron concentration using spectrophotometry because of its retrospective nature. Second, all measurements for the LMR were obtained in the right lobe of the liver to avoid motion-related artifact. Because the pathologic specimens were obtained at surgery for an HCC, histologically sampled areas did not completely correspond to radiologically sampled areas. A prospective study with a substantially larger sample is needed to further validate our findings.

In conclusion, DW-EPI (especially, T2-weighted SSEPI) was sensitive to hepatic iron, and might be a more useful sequence for quantifying mild degree of hepatic iron stores in patients with chronic viral hepatitis.
