# Correction: Quantification of Hepatic Iron Concentration in Chronic Viral Hepatitis: Usefulness of T2-weighted Single-Shot Spin-Echo Echo-Planar MR Imaging

**DOI:** 10.1371/annotation/b1c45b8e-79a5-48a3-a464-d8b339bc1a7e

**Published:** 2012-07-10

**Authors:** Tatsuyuki Tonan, Kiminori Fujimoto, Aliya Qayyum, Takumi Kawaguchi, Atsushi Kawaguchi, Osamu Nakashima, Koji Okuda, Naofumi Hayabuchi, Michio Sata

There were errors in affiliations 1 and 7. The correct affiliations are:

1 Department of Radiology, Kurume University School of Medicine, Kurume, Fukuoka, Japan,

7 Department of Surgery, Kurume University School of Medicine, Kurume, Fukuoka, Japan. 

